# The Impact of Bevacizumab and Chemotherapy on Quality of Life in Metastatic Colorectal Cancer Patients

**DOI:** 10.3390/healthcare11040591

**Published:** 2023-02-16

**Authors:** Diana Cornelia Moisuc, Mihai Vasile Marinca, Andreea Mihaela Matei, Larisa Popovici, Petru Cianga

**Affiliations:** 1 Immunology Department, “Grigore T. Popa” University of Medicine and Pharmacy, 700115 Iasi, Romania; 2 Oncology Department, “Grigore T. Popa” University of Medicine and Pharmacy, 700115 Iasi, Romania; 3 Oncology Department, Regional Institute of Oncology, 700483 Iasi, Romania; 4 Immunology Department, “St. Spiridon” Hospital, 700111 Iasi, Romania

**Keywords:** bevacizumab, chemotherapy, metastatic colorectal cancer, quality of life

## Abstract

Health-related quality is of life of great importance in cancer care. This prospective study aimed to evaluate the impact of chemotherapy and bevacizumab on the activities of daily living, cancer symptoms, and general well-being in 59 metastatic colorectal cancer patients. We gathered information using the EORTC QLQ-C30 and QLQ-CR29 questionnaires. The paired sample *t*-test, MANOVA test, and Pearson’s correlation test were used to analyze the presence of significant differences in mean scores before and after 6 months of treatment. The results revealed significant differences in the functioning and symptoms that influence patients’ quality of life after 6 months of treatment: increased pain (*p* = 0.003), nausea and vomiting (*p* = 0.003), diarrhea (*p* = 0.021) and decreased appetite (*p* = 0.003). At the same time, there were several aspects that improved the quality of life. Increases in emotional function (*p* = 0.009), cognitive function (*p* = 0.033), and perception of body image (*p* = 0.026) were observed after 6 months of treatment. Elderly patients reported a higher frequency of stools (*p* = 0.028), and young patients had increased concerns about body perception (*p* = 0.047). Assessing the quality of life of metastatic colorectal cancer patients is an important way to identify and treat symptoms related to both cancer and therapy by establishing a holistic care plan and implementing measures to increase the quality of life.

## 1. Introduction

Colorectal cancer (CRC) is the third most common malignancy and the second leading cause of cancer death worldwide. In 2020, 1.9 million new cases and 0.9 million deaths were estimated [1,2,3]. The most important factor in reducing mortality, preventing metastasis, and improving prognosis and quality of life is early diagnosis, but unfortunately, CRC symptoms occur in advanced stages [4]. The main treatment methods are surgery, adjuvant or palliative chemotherapy, targeted therapy, and, more recently, immunotherapy. These treatments can have long- and short-term effects and complications that, together with the symptoms resulting from neoplasia and associated comorbidities, lead to impaired quality of life in patients with metastatic colorectal cancer. The efficacy of therapeutic intervention in cancer is measured by assessing the response rate, progression-free survival, or overall survival, but in the case of metastatic neoplasia, the benefits should also be assessed by validated questionnaires assessing the impact on patients’ quality of life, in addition to traditional goals.

In recent decades, assessing and improving the quality of life has become an important goal in cancer research, for both the clinician and the patient [5,6,7], and it is defined as the well-being of a person or society in terms of health and happiness [6]. This includes both physical and psychological health, the level of functioning, social relationships, and personal beliefs. Quality of life has become a standard “end-point” in randomized clinical trials and contributes to the therapeutic decision by presenting information from the patient’s perspective. Factors such as the presence and location of metastases, the location of the primary tumor, treatment, colostomy, age, performance status, education level, financial resources, and family and social support all influence the quality of life of cancer patients [8,9].

Previous studies targeting patients with colorectal cancers have highlighted the impact of various therapeutic approaches on various aspects of life quality. Cancer patients often suffer from depression, and Gray et al. discovered that patients with metastatic colorectal cancer (mCCR) present an increased risk of depression, which is not influenced by the type of treatment received [10]. Chemotherapy, among many other side effects, can lead to impaired cognitive function [11,12]. Two of the most frequently used regimens associated with bevacizumab include Irinotecan and Oxaliplatin. Irinotecan-based chemotherapy leads to gastrointestinal disturbances and alopecia [13], while Oxaliplatin mostly causes peripheral neuropathy, hypersensitivity reactions, and myelosuppression [14]. A particular aspect influencing the quality of life in colorectal cancer patients is a colostomy, but surprisingly, the psychosocial impact or post-procedural complications appear unaffected by age [15]. On the other hand, the subjective perception of quality of life in colorectal cancer patients is influenced by gender [16,17,18].

Furthermore, quality of life has been studied as an independent predictor for survival and response to treatment. Braun et al. observed that a 10-point increase in quality of life from baseline was associated with a 7% reduction in mortality risk in patients with CRC [19]. Similar results have been obtained in bladder [20] or head and neck cancers [21]. At the same time, patients’ quality of life also influences their adherence to treatment, which ultimately affects survival.

Although the quality of life of patients is a subject of immense interest, especially in the case of colorectal cancer patients, in terms of symptoms [6,22,23,24,25,26], age [15,27,28,29,30,31], and treatment [32,33,34,35,36,37], little is known about the influence of the adverse effects of chemotherapy combined with those given by bevacizumab on the quality of life of patients with metastatic colorectal cancer. Marques et al. focused not only on health-related quality of life but also on the patient-reported outcome in colorectal cancer patients and concluded that a progressive negative impact on these two factors was induced by regimens including cetuximab, while those including bevacizumab were closer to the baseline [38].

The aim of this prospective study was to assess the quality of life of patients with metastatic colorectal cancer at baseline, and the changes that occur after 6 months of treatment with bevacizumab and chemotherapy, and to compare our results with other similar studies that enrolled patients with various characteristics. When looking at studies that evaluated the quality of life of colorectal cancer patients receiving bevacizumab, several study directions could be identified. Some focused on patients who received irinotecan, with or without bevacizumab [39,40], some on elderly patients receiving chemotherapy with or without bevacizumab [41], and some on patients receiving cetuximab with chemotherapy versus bevacizumab with chemotherapy [38,42]. The design of our study was closer to the one proposed by Ward et al. who compared chemotherapy and bevacizumab, but again in elderly patients, who were further divided into two groups, depending on the ECOG performance status [43]. Our study group included both elderly and younger patients, with an age limit set at 65 years. Furthermore, most of the studies that evaluated the quality of life of colon cancer patients focused on other symptoms such as anxiety [10,44,45], mental status [24], fatigue [25], colostomy [6,22], and other therapies, including surgical techniques [31,37]. However, we were rather interested in determining how the addition of bevacizumab and chemotherapy regimens influences various parameters usually considered when evaluating the life quality impairment, such as physical functioning, role functioning, emotional functioning, cognitive functioning, social functioning, fatigue, nausea/vomiting, pain, dyspnea, insomnia, and appetite loss.

## 2. Materials and Methods

### 2.1. Data

We conducted a prospective study that included patients undergoing treatment at the Regional Institute of Oncology in Iași, who met the following inclusion criteria: signed informed consent, age over 18 years, histopathological diagnosis of colorectal cancer, stage IV according to the TNM classification, Eastern Cooperative Oncology Group (ECOG) 0–2 performance status, life expectancy of at least 6 months, adequate bone marrow, renal and liver function, and biological therapy with bevacizumab. The exclusion criteria were known hypersensitivity to bevacizumab or chemotherapy, chronic heart failure > NYHA class II, acute ischemic disease, other conditions that could affect the patient’s compliance with the investigator’s decision, pregnancy, and breastfeeding.

In the initial group, 88 patients were included, but 29 were lost to follow-up and were excluded from the statistical analysis. Finally, the analysis was performed on a group of 59 patients with complete data both at the beginning and after 6 months of treatment.

Patients were scheduled to start treatment with bevacizumab 7.5 mg/kg every 3 weeks or 5 mg/kg every 2 weeks, along with a standard chemotherapy protocol, depending on their treating physician. The options were oxaliplatin-based chemotherapy (*n* = 39; CapeOX or mFOLFOX 6), irinotecan-based chemotherapy (*n* = 17; XELIRI or FOLFIRI), or fluoropyrimidine-based chemotherapy (*n* = 3; Capecitabine alone or de Gramont).

### 2.2. Measures

For the assessment of health-related quality of life, the European Organization for Research and Treatment of Cancer Quality of Life Questionnaire C30 (EORTC QLQ-C30 V3.0) and colorectal-specific questionnaire (EORTC QLQ-CR29 V2.1) were used [46]. Questionnaires were self-completed by patients before the first dose of treatment and 6 months after the treatment initiation.

EORTC QLQ-C30 is a specific questionnaire used as a basic tool in the assessment of the quality of life of cancer patients. It includes 30 questions ordered in 5 functional scales: physical, emotional, cognitive, social functionality, and social roles; 3 symptomatic scales: fatigue, pain, nausea/vomiting, a general condition scale; and 6 symptoms: dyspnea, decreased appetite, insomnia, constipation, diarrhea, and financial difficulties. The EORTC QLQ-CR29 questionnaire is a specific module for colorectal cancer and contains 29 questions arranged in 4 functional scales: anxiety, weight, body image, sexual functionality, and 18 symptomatic scales such as abdominal pain, flatulence, meteorism, fecal incontinence, urinary incontinence, dysuria, stoma care, alopecia, and change in taste.

We evaluated the results of the questionnaires according to the instructions of the Scoring Manual, third edition [46,47]. Most items in the two questionnaires were coded and scored from 1 to 4, namely: “not at all”, “a little”, “quite a bit” and “very much”. The scoring principle of these scales was the same in all cases: the estimation of the average of the items that contributed to the scale (raw score) and, subsequently, the linear transformation, to standardize the raw score so that scores range from 0 to 100 based on well-defined formulas. A high score for a given scale represents a good quality of life, whereas a high score for a given symptom represents a poor quality of life as a result of that symptom.

### 2.3. Statistical Analytical Strategy

For statistical analysis, we used the SPSS v.16.0 software (SPSS Inc., Chicago, IL, USA). Qualitative and quantitative variables were characterized by frequency, mean, median and standard deviation, to describe the baseline characteristics of the study population.

The paired sample *t*-test was used to compare the means of quality of life measures assessed at two different time points (baseline and after 6 months of treatment) for each scale or symptom, with *p* < 0.05 indicating statistical significance. The MANOVA test was used to assess whether there were differences in quality of life of patients with metastatic CRC according to age, gender, chemotherapy protocol, primary tumor surgery, colostomy, or associated comorbidities, and Kaplan–Meier was used to estimate progression free survival (PFS). We checked the assumptions for MANOVA. To test the normal distribution, we used the Shapiro–Wilk test. The independence was checked by plotting a scatterplot matrix for each group, while for equal variance, we used Levene’s test.

Pearson’s correlation test was used to assess the relationship between global health status, certain scales, and symptoms and PFS. Cohen’s D method was used to calculate the *t*-test effect size. Partial Eta Squared was used for MANOVA and Eta Squared test to analyze the association between global health status, both at the beginning and after 6 months of treatment, and the tumor response according to RECIST 1.1 [48].

## 3. Results

A total of 59 consecutive patients with metastatic CRC, who received concomitant chemotherapy with bevacizumab between May 2019 and September 2021, were included in the study. The median age of the patients was 61 (37, 82) years; 39 patients were under 65 years of age. More than half of the participants were male (*n* = 32, 54%). Associated comorbidities have been classified into the following categories: heart disease (e.g., heart failure, atrial fibrillation, hypertension, aortic or mitral regurgitation), diabetes mellitus, pulmonary disease (e.g., chronic obstructive lung disease and pulmonary emphysema), and kidney failure. Most of the tumors (80%) were located on the descending colon. Mutations in RAS (KRAS, NRAS) genes were present in 39 cases. The liver was the most common site for metastasis (46%), and 24 patients had more than one metastatic site. The median PFS was 10 months in the entire study population. The tumor response was evaluated according to the RECIST 1.1 criteria and was obtained in the case of 41 patients (partial response and stable disease). No patient obtained a complete response, and 18 patients showed progressive disease after treatment. The baseline patient and disease characteristics are summarized in Table 1.

**Figure 1:**
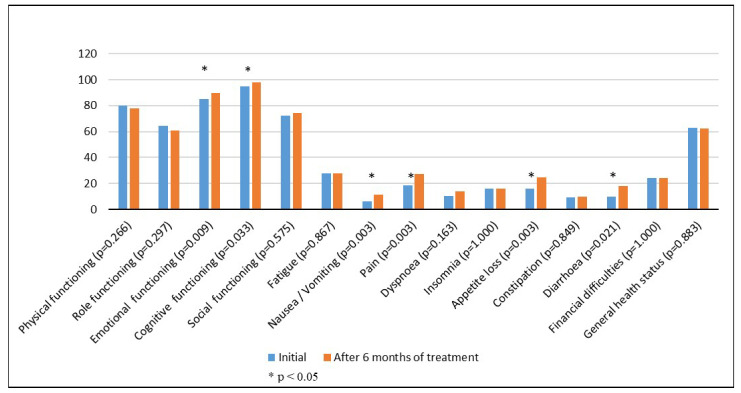
European Organization for Research and Treatment of Cancer QLQ-C30 questionnaire.

**Figure 2:**
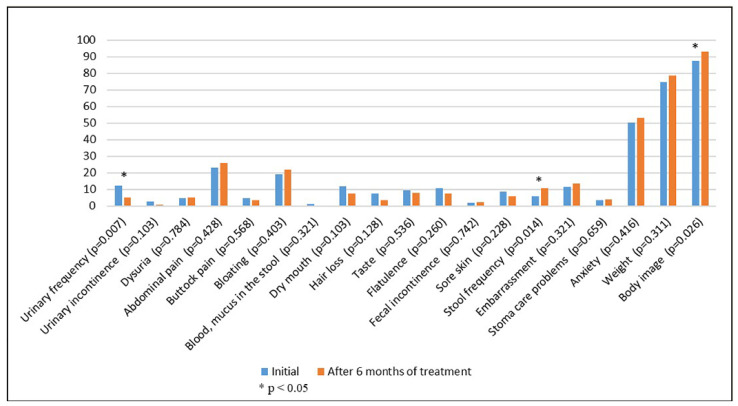
European Organization for Research and Treatment of Cancer QLQ-CR29 questionnaire.

When quality of life was compared at the beginning and after 6 months of treatment, an increase in the intensity of any of the following symptoms was observed: pain (*p* = 0.003), decreased appetite (*p* = 0.003), nausea and vomiting (*p* = 0.003), diarrhea (*p* = 0.021), and an improvement in emotional function (*p* = 0.009), cognitive function (*p* = 0.033), and perception of body image (*p* = 0.026). There was also a tendency to improve the social component but without reaching the statically significant threshold (Table 2, Figure 1 and Figure 2). Cognitive function had the highest score, both at the initial evaluation and after 6 months of treatment. The overall health scale had a score of 62.71 at the initial assessment and 62.43 at the final assessment (*p* = 0.883), and the most commonly reported symptom was abdominal pain.

To assess if the quality of life differs between young and elderly patients, we divided the group into two age categories: under 65 and over or equal to 65. The second category included 20 patients. The result of the MANOVA analysis showed that elderly patients experienced hair loss of higher intensity at 6 months, which was statistically significant compared to that of young patients (*p* = 0.012, η_p_^2^ = 0.123). Elderly patients also reported a higher frequency of stools at the start of treatment (*p* = 0.028, η_p_^2^ = 0.082). In contrast, young patients expressed increased concern about body perception at 6 months after treatment (*p* = 0.047, η_p_^2^ = 0.067).

Female patients had better cognitive function at the start of treatment compared to men (*p* = 0.05, η_p_^2^ = 0.066), they had an increase in stool frequency after 6 months of treatment (*p* = 0.024, η_p_^2^ = 0.086), and they showed an increase in their level of anxiety at the second evaluation (*p* = 0.046 η_p_^2^ = 0.068). Regarding body image, female patients had a low level both at initiation and 6 months after treatment (*p* = 0.35, η_p_^2^ = 0.075 and *p* = 0.45, η_p_^2^ = 0.069, respectively). Compared to women, men reported a higher intensity of sore rectal or colostomy skin, both at the initiation of treatment and after 6 months (*p* = 0.033, η_p_^2^ = 0.077 and *p* = 0.012, η_p_^2^ = 0.105, respectively), and had greater financial difficulties 6 months after treatment (*p* = 0.037, η_p_^2^ = 0.074).

Six months after treatment, patients who underwent excision of the primary tumor showed improvement in social function (*p* = 0.022, η_p_^2^ = 0.088), improvement in the intensity of abdominal pain (*p* = 0.006, η_p_^2^ = 0.123), and pain in the anal area (*p* = 0.007, η_p_^2^ = 0.120).

Patients with colostomy reported an increased level of flatulence at the beginning of treatment (*p* = 0.022, η_p_^2^ = 0.089), increased intensity of sore skin both at the beginning and after 6 months of treatment (*p* < 0.001, η_p_^2^ = 0.210, *p* = 0.003, η_p_^2^ = 0.148), and an increased level of embarrassment regarding the intestinal transit (*p* < 0.001, η_p_^2^ = 0.419).

Patients who had associated comorbidities, regardless of their type, had an increased level of anxiety 6 months after treatment, but the difference did not reach the level of statistical significance compared to patients without comorbidities (*p* = 0.56, η_p_^2^ = 0.061). Regarding the chemotherapy regimen associated with bevacizumab therapy, there were no statistically significant differences in changes in patients’ quality of life. Indeed, chemotherapy regimens give different adverse effects, and the quality of life can be influenced differently, but in our study, we did not have statistically significant differences.

The results show that patients who received adjuvant chemotherapy had a lower level of cognitive (*p* < 0.001, η_p_^2^ = 0.233) and social function (*p* = 0.016, η_p_^2^ = 0.098), an increased level of dyspnea (*p* = 0.004, η_p_^2^ = 0.136), bloating (*p* = 0.032, η_p_^2^ = 0.078), and fecal incontinence (*p* = 0.041, η_p_^2^ = 0.071) at the beginning of treatment with bevacizumab. The location of the primary tumor (left colon versus right colon) did not influence patients’ quality of life.

A statistically significant correlation was observed between the physical functional scale and age (r = −0.275, *p* = 0.035), body image scale, and age (r = 0.341, *p* = 0.008) at the initiation of the treatment. Additionally, the physical functional scale was correlated with the global health status scale (r = 0.539, *p* < 0.001), emotional functional scale (r = 0.399, *p* = 0.002), cognitive functional scales (r = 0.272, *p* = 0.037), and social functional scale (r = 0.446, *p* < 0.001) after 6 months of treatment. Furthermore, a statistically significant correlation was found between the global health status scale and financial difficulties (r = −0.345, *p* = 0.007). No correlation was found between the global health status scale and age and between global health status, scales, symptoms, and progression-free survival. Moreover, we performed the Eta squared test to find out the association between the global health status, both at the beginning and after 6 months of treatment, and the tumor response. We obtained a weak association between the two variables (η^2^ = 0.0216).

## 4. Discussion

In the current study, we investigated the effects of chemotherapy combined with bevacizumab on the functionality and quality of life of patients diagnosed with metastatic colorectal cancer. We chose the EORTC-QLQ C30 and EORTC QLQ-CR29 questionnaires, as they are probably the most valid and commonly used tools to assess the quality of life in patients with cancer [46,47,49,50]. These questionnaires focus on assessing the patient’s ability to perform daily activities, justifying their use in both routine practice and clinical trials.

The results of the study showed that, after 6 months of treatment, patients displayed a markedly decreased appetite, increased nausea, vomiting, and diarrhea—symptoms associated with the administration of chemotherapy regimens in metastatic colorectal cancer [13,51,52]. Increased pain levels were also observed, which may be associated with disease progression, neuropathic pain due to oxaliplatin treatment [53], or a decrease in the psychological component [54,55]. Conversely, an improvement in emotional, cognitive functioning, and body image perception was observed.

Emotional function was assessed by measuring the level of concern, anxiety, and depression. Factors influencing the occurrence or increase in anxiety in cancer patients are the female gender, young patients, and symptoms such as fatigue, dyspnea, and anorexia. Gray et al. reported that patients with mCCR have an increased risk of depression, which is further increased among those who receive treatment, regardless of its type [10]. Another study showed that patients who had surgery alone had lower levels of depression and anxiety compared to patients who also received chemotherapy or radiotherapy [45]. The results of our study are in contradiction with the mentioned studies. We have shown that after 6 months of treatment, the emotional function improved by five points. This improvement may be associated with an increased level of social, family, and healthcare support [56,57]. Among patients with associated comorbidities, regardless of their type, anxiety levels were increased at the 6-month post-treatment assessment, but without reaching the threshold of statistical significance.

Cognitive impairment is a common side effect experienced to varying degrees by approximately 75% of cancer patients [58] and can have a significant impact on quality of life, affecting attention, concentration, spatial-visual ability, verbal ability, and memory. Several studies have shown that the main factor in impaired cognitive function is the administration of chemotherapy [11,12]. The mechanism by which cognitive impairment occurs is not fully understood but may be associated with chronic inflammation, oxidative stress, increased toxicity of the chemotherapy regimen (oxaliplatin-induced neurotoxicity), radiotherapy, genetic susceptibility, or psychotropic medication administration [59]. The results of our study showed that cognitive function improved by four points after 6 months of treatment. Male patients showed a significantly higher level of cognitive impairment at treatment initiation. Age was not a risk factor. Patients over 65 years of age showed similar levels of cognitive function to young patients both before and after 6 months of treatment. Our results are supported by the fact that cognitive function is correlated with the level of anxiety and depression [60] which, in this study, had a low level.

Body image is defined as the perception, satisfaction, and attitude of a person towards his or her body, which is an important aspect of oncology, especially in patients undergoing mutilating surgery (radical mastectomy, colostomy colectomy, or urostomy cystectomy) [6]. A patient’s response to cancer and treatment decision can be influenced by many variables, including physical factors. Potential changes in physical appearance, function, or body integrity are important aspects of treatment decisions from a patient’s point of view. Quality of life is defined as the state of health perceived by the patient in relation to the physical, psychological, social, and spiritual appearance. A meta-analysis showed that patients with colostomy following colorectal cancer surgery have a low quality of life and problems related to complications, as care of the colostomy negatively influences the quality of life [22]. The most common colostomy-related problems were sexual problems, depression, abdominal meteorism, constipation, dissatisfaction with physical appearance, and travel difficulties. The factors that influenced the perception of colostomy were age, gender, and time since colostomy. Another study concluded that age does not influence the occurrence of complications related to performing a colostomy. Patients over 70 years old who had colostomy did not display statistically significant differences from younger patients in terms of psychosocial impact or post-procedural complications [15], but, regardless of the presence or absence of colostomy, geriatric patients have a higher rate of perioperative mortality. 

The results of our study show that, after 6 months of treatment, body image perception improved in the general population, which is consistent with the results of another study showing that patients experience changes in their quality of life over time and they adapt to the new conditions and body image perceptions [61]. When analyzed by gender subgroups, female patients showed a low level of body image perception, both at diagnosis and at 6 months of treatment. The presence of the colostomy produced several symptoms such as flatulence, sore skin around the stoma, and embarrassment regarding bowel movements. However, the needs of patients with a stoma are more complex, including psychological, social, and spiritual aspects in addition to the physical, which have not been assessed in this study and are difficult to quantify.

Our study shows that age does not influence the overall quality of life of patients in the study group, either at baseline or after 6 months of treatment with chemotherapy and bevacizumab. In the geriatric population, a person’s quality of life correlates with their degree of mobility, autonomy, and preserved intellectual and cognitive function; however, comorbidities and geriatric syndromes such as the risk of falls, osteoporosis, urinary incontinence, and dementia are frequently present, leading to a decrease in physiological reserves and quality of life. Little is known about the quality of life, functional status, and burden of symptoms in vulnerable geriatric patients with mCCR receiving palliative chemotherapy. The elderly population is generally treated based on data extrapolated from selected younger cohorts, but the same therapeutic methods may be less effective and more toxic in elderly people with comorbidities. In addition, many elderly patients with cancer tend to prioritize improving or maintaining quality of life over prolonged survival [62]. One study has shown that reducing the dose of chemotherapy does not affect the quality of life in elderly patients who do not meet the criteria for standard treatment [27]. McCombie et al. have shown that, for patients over 80 years of age with colorectal cancer, autonomy, mobility, and avoidance of stoma and surgery are more important than prolonging survival [30], but the quality of life can be improved in elderly patients after surgery, even for vulnerable patients [28].

The results of several studies have shown that gender differences may influence the perception of quality of life in patients with colorectal cancer [16,17,18]. Impairment of cognitive function and sexual problems reduce quality of life, with a greater impact among men, while body image, abdominal pain, and mucosal dryness have led to a lower quality of life among female patients. A longitudinal study involving 75 patients with rectal cancer concluded that one year after surgery, female patients had a poorer overall condition and impaired quality of life due to fatigue, weight loss, and future prospects. On the other hand, for men, defecation problems and financial difficulties had a higher share [63]. In addition to the health impairment, the effects of cancer and treatments are also seen in the professional field, leading to a decrease in productivity at the workplace, retirement, or sick leave, which ultimately lead to social isolation and depression. This was confirmed by the results of a study showing that men had more difficulties carrying out daily activities, while the quality of life among women was affected by physical symptoms, social isolation [64], and anxiety [44]. The results of our study are consistent with the data in the literature. After 6 months of treatment, men had more important financial difficulties while women showed an increased level of anxiety and impaired body perception.

Chemotherapy, despite improved overall survival, produces toxicity that affects the quality of life. When comparing different treatment regimens for metastatic colorectal cancer, no difference in impairment of quality of life was observed, but when adding targeted therapy, bevacizumab was observed to be better tolerated compared to cetuximab [65]. Oxaliplatin-based chemotherapy most commonly causes peripheral neuropathy, hypersensitivity reactions, and myelosuppression [14], while irinotecan causes gastrointestinal disturbances and alopecia [13]. The addition of bevacizumab to the chemotherapy regimen does not produce changes in the perception of quality of life in patients with colorectal cancer [39,40], but, according to the results of a study by Canevari et al., in patients with ovarian cancer, the addition of bevacizumab resulted in a 10-point decrease in quality of life [66]. In this study, the chemotherapy regimen combined with bevacizumab therapy did not produce statistically significant differences in patients’ quality of life.

The results of our study have practical Implications for clinicians. Symptoms influencing the quality of life identified after 6 months of therapy with bevacizumab and chemotherapy can be treated accordingly, improving the quality of life of patients with metastatic colorectal cancer and the rate of compliance with treatment. Appetite may be influenced by non-pharmacological therapies such as nutritional counseling, increased physical activity, and pharmacological therapies such as megestrol acetate, corticosteroid therapy, or omega-3 fatty acids [67,68]. The emetogenic syndrome is commonly seen after chemotherapy and requires a full assessment including the frequency, duration, and time of onset of nausea or vomiting. Numerous international guidelines have been developed for the proper prophylaxis of emesis induced by chemotherapy or radiotherapy. The most commonly recommended antiemetic agents are 5-hydroxytryptamine receptor antagonists, neurokinin 1 receptor antagonists, corticosteroids, and benzodiazepines for emetogenic psychogenic syndrome [69,70]. 

Chemotherapy-induced diarrhea causes a high degree of morbidity and mortality if not treated appropriately. Management is complex and involves careful assessment of the patient, hydro-electrolytic imbalances, and possible bacterial or viral associations. Loperamide is the first-line medication in the treatment of chemotherapy-induced diarrheal stools. Codeine, octreotide, atropine, antibiotics, and the correction of hydro-electrolyte imbalances may also be recommended where appropriate [71]. Pain is one of the most common and fearsome symptoms in patients with cancer. Between 30% and 50% of patients receiving anticancer therapies and more than 70% of patients with advanced stages of the disease complain of this symptom [72]. The onset of pain is tiered, according to the level of intensity recounted by the patient. The WHO analgesia scale classifies pain relieving medication in three different ways: non-opioids (non-steroidal anti-inflammatory drugs and paracetamol), weak opioid analgesics (tramadol), and strong opioid analgesics (morphine), with which coanalgesics can be associated: antidepressants (tricyclics, selective serotonin reuptake inhibitors, serotonin and noradrenaline reuptake inhibitors), neuroleptics, anticonvulsants, steroids, and benzodiazepines [54,55].

By the means of specific prophylactic and interventional measures, the quality of life of patients with metastatic colorectal cancer undergoing treatment with chemotherapy and bevacizumab can be improved, requiring a multidisciplinary approach to cancer patient management.

## 5. Conclusions

The results of the study conclude that bevacizumab and chemotherapy as a cancer treatment modality contribute to the aggravation of symptoms, affecting patients’ quality of life. After 6 months of treatment, the level of pain, nausea, vomiting, and diarrhea increased. Additionally, the decrease in appetite was more pronounced. However, there were several aspects that improved quality of life. A statistically significant increase in emotional function, cognitive function, and perception of body image was observed after 6 months of treatment. These results are contradictory to the data obtained from other studies. The patients’ age and the chemotherapy regimen associated with bevacizumab did not influence quality of life in the present study. The quality-of-life assessment of patients with metastatic colorectal cancer should be considered when choosing a therapeutic plan, and repeated assessments are required during treatment to correctly identify and treat the symptoms affecting the patients’ quality of life.

## Figures and Tables

**Table 1:** Patient and disease characteristics.

Characteristic	Frequency	Percent
Median age	61 (37, 82)	
Gender		
Male	32	(54)
Female	27	(46)
Comorbidities		
Cardiovascular ^1^	24	(41)
Diabetes mellitus	6	(10)
Pulmonary ^2^	3	(5)
Renal	2	(3)
Primary tumor location		
Left colon	47	(80)
Right colon	12	(20)
Stage at diagnosis		
Metastatic	47	(80)
Non-metastatic	12	(20)
Primary tumor resection	42	(71)
Colostomy	14	(24)
RAS status		
Wild type	17	(29)
Mutant	39	(66)
KRAS mutation	33	(56)
NRAS mutation	6	(10)
Not tested	3	(5)
Chemotherapy regimen		
Oxaliplatin-based	39	(66)
Irinotecan-based	17	(29)
Fluorouracil/Capecitabine-based	3	(5)
Metastases		
Liver	27	(46)
Lung	4	(7)
Peritoneal	4	(7)
Multiple	24	(41)

^1^ heart failure, atrial fibrillation, hypertension, aortic or mitral regurgitation; ^2^ chronic obstructive lung disease, pulmonary emphysema.

**Table 2:** Quality of life at baseline and after 6 months of treatment according to EORTC QLQ-C30 and EORTC QLQ-CR29.

	Initial		After 6 Months		*p*	Effect Size
	Median	Standard Deviation	Median	Standard Deviation		
Physical functioning	79.77	10.20	77.97	11.09	0.266	0.170
Role functioning	64.41	21.09	61.02	16.55	0.297	0.179
Emotional functioning	84.89	13.17	89.55	8.97	0.009	0.414
Cognitive functioning	94.63	10.46	98.02	6.25	0.033	0.393
Social functioning	72.32	15.96	74.01	15.86	0.575	0.107
Fatigue	27.68	13.27	28.06	12.44	0.867	0.029
Nausea/Vomiting	5.93	10.61	11.58	13.58	0.003	0.464
Pain	18.64	15.80	27.12	19.05	0.003	0.484
Dyspnea	10.17	16.67	14.12	17.72	0.163	0.230
Insomnia	15.82	20.85	15.82	21.75	1.00	9.4 × 10^−6^
Appetite loss	15.82	20.85	24.86	17.05	0.003	0.474
Constipation	9.04	18.39	9.60	18.62	0.849	0.030
Diarrhea	9.60	17.56	18.08	20.82	0.021	0.440
Financial difficulties	24.29	25.39	24.29	23.83	1.00	1.2 × 10^−5^
Global health status	62.71	12.31	62.43	12.31	0.883	0.023
Urinary frequency	12.43	18.97	5.08	10.83	0.007	0.475
Urinary incontinence	2.82	9.36	0.56	4.33	0.103	0.310
Dysuria	4.52	13.06	5.08	13.58	0.784	0.042
Abdominal pain	23.16	19.82	25.99	20.59	0.428	0.140
Buttock pain	4.52	11.50	3.39	11.89	0.568	0.097
Bloating	19.21	20.71	22.03	19.18	0.403	0.141
Blood, mucus in the stool	0.85	3.69	0.28	2.16	0.321	0.187
Dry mouth	11.86	16.09	7.34	13.93	0.103	0.300
Hair loss	7.34	18.63	3.39	10.16	0.128	0.263
Taste	9.60	16.43	7.91	14.30	0.536	0.110
Flatulence	10.73	15.70	7.34	15.24	0.260	0.219
Fecal incontinence	1.69	7.38	2.26	10.47	0.742	0.062
Sore skin	8.47	15.89	5.65	12.61	0.228	0.197
Stool frequency	5.65	10.99	10.45	13.80	0.014	0.385
Embarrassment	11.30	17.07	13.56	16.51	0.321	0.135
Stoma care problems	3.39	10.16	3.95	10.87	0.659	0.054
Anxiety	50.28	22.63	53.11	17.63	0.416	0.139
Weight	74.58	27.22	78.53	21.22	0.311	0.162
Body image	87.57	16.38	93.03	13.19	0.026	0.367

## Data Availability

The datasets generated and analyzed during the current study are available from the corresponding author and can be shared with the journal for review if needed.
